# Application of transesophageal echocardiography for localization in totally implantable venous access port implantation through subclavian approach in children

**DOI:** 10.1002/clc.23518

**Published:** 2020-11-25

**Authors:** Shujun Yang, Xiangru Kong, Lifei Liu, Ying Xu, Jun Zhang

**Affiliations:** ^1^ Department of Anesthesiology Chongqing Medical University Affiliated Children's Hospital Chongqing China; ^2^ Department of Surgical Oncology Chongqing Medical University Affiliated Children's Hospital Chongqing China; ^3^ Ministry of Education Key Laboratory of Child Development and Disorders Chongqing China; ^4^ National Clinical Research Center for Child Health and Disorders Chongqing China; ^5^ China International Science and Technology Cooperation Base of Child development and Critical Disorders Chongqing China; ^6^ Childrens Hospital of Chongqing Medical University Chongqing China; ^7^ Chongqing Key Laboratory of Pediatrics Chongqing China

**Keywords:** chest X‐ray, subclavian vein, superior vena cava, totally implantable venous access port, transesophageal echocardiography

## Abstract

A totally implantable venous access port (TIVAP) is important in children who need intravenous infusion for a long time. A number of studies have shown methods for locating the tip of the TIVAP catheter. To explore whether transesophageal echocardiography (TEE) can be used to accurately locate the TIVAP catheter tip through a subclavian approach and to improve the rate of correct TIVAP catheter placement and reduce complications of TIVAP placement. In 36 children who needed TIVAP implantation surgery, we used real‐time TEE guidance to place the catheter tip around the crista terminalis. In all children, chest X‐rays were used to figure out whether the catheter tip as localized by TEE was within the T5‐T7 segment. Then, we compared the length of the catheter calculated by the height formula and the actual catheter length applied under TEE guidance. The medical records, surgical details, nursing records, and recorded complications were collected during the follow‐up. The success rate of TIVAP implantation was 100% in all enrolled patients and no hemopneumothorax or pinch‐off syndrome occurred. Compared with TEE, chest X‐ray showed a coincidence rate of 80.56% in correctly detecting the TIVAP catheter tip locate. The height‐derived catheter length (11.0 [9.6, 11.8]) cm and the TEE‐derived catheter length (10.0 [9.3, 10.8]) cm were significantly different (p < .001). TEE can be used to guide TIVAP catheter positioning through a left subclavian approach in children accurately and successfully and more accurate than chest X‐ray and height calculation formula.

**Abbreviations**
CCSG: Children's Cancer Study Group
CVC: central venous catheter
EMR: electronic medical records
HIS: hospital information system
IQR: interquartile range
PLT: platelet count
RA: right atrium
SCV: subclavian vein
SVC: superior vena cava
TEE: transesophageal echocardiography
TIVAP: totally implantable venous access port
TTE: transthoracic echocardiography

## 1 INTRODUCTION

The use of a totally implantable venous access port (TIVAP) is essential in children with malignant tumors who need long‐term infusion therapy. The TIVAP is widely used in the clinic because of its advantages of long‐term retention, easy management, and low‐infection rate. The American Multi‐Center Children's Oncology Research Group (Children's Cancer Study Group, CCSG) also recommended it as the first choice for chemotherapy in children with hematological tumors in 1992.^1^ Inaccurate localization of the catheter will lead to severe complications, such as cardiac tamponade and cardiac perforation.^2^, ^3^ Since the consequences of catheter misplacement could be hazardous and sometimes lethal to the patient,^4^, ^5^ correctly detecting the catheter position is very important. Most studies have recommended the subclavian vein (SCV) as the first choice for TIVAP implantation in children because of its high comfort and low‐postoperative complications.^6^, ^7^, ^8^, ^9^

In the past, chest X‐ray was used to locate the tip of the catheter. However, chest X‐rays may lead to catheter malpositioning,^10^, ^11^ sometimes may cause fatal hemopneumothorax, and multiple exposures are needed to locate the catheter during or after surgery. Transesophageal echocardiography (TEE) can be used to dynamically observe images of the chambers (left atrium, right atrium [RA], left ventricle, and right ventricle) and surrounding blood vessels (superior vena cava [SVC], postcava, aorta, pulmonary artery, and pulmonary vein) in real time. Some studies have shown the usefulness of TEE in guiding central venous catheter (CVC) placement^12^, ^13^; however, studies assessing the positioning of TIVAP catheters via TEE are scarce. Furthermore, the two‐dimensional images of TEE can be used to accurately determine whether the guide wire has entered the SVC to avoid fatal hemopneumothorax caused by incorrect insertion of the vascular expansion sheath.^6^ In this prospective study, we tested the hypotheses that TEE could be used to guide catheter cannulation and would both improve the rate of correct intravenous infusion port catheter positioning and reduce the incidence of related complications in children.

## 2 MATERIAL AND METHODS

This study was approved by the Institutional Review Board for Human Studies at the Children's Hospital of Chongqing Medical University (2019–230). Prior written informed consent was provided by the child or the child's guardians. The child or child's guardian signed the informed consent and participated in this study.

### 2.1 Patients

From November 2019 to February 2020, 36 patients undergoing TIVAP implantation surgery at the Children's Hospital of Chongqing Medical University were included in this prospective study. The inclusion criteria were age from 3 months to 18 years, weight of more than 5 kg and no skin infection in the surgical area or other parts of the body. The exclusion criteria were absence of sinus rhythm, upper gastrointestinal bleeding, esophageal disease, pharyngeal space‐occupying lesions, SVC obstruction syndrome, blood coagulation dysfunction, neutrophils0.5*10^9^/L, and PLT50*10^9^/L.

### 2.2 Surgical technique

All patients underwent standardized anesthesia, and mechanical ventilation was performed after the induction of anesthesia and completion of endotracheal intubation. The head was kept low at 15, and the bilateral neck, chest, and shoulders were fully exposed after routine disinfection.

**FIGURE 1:**
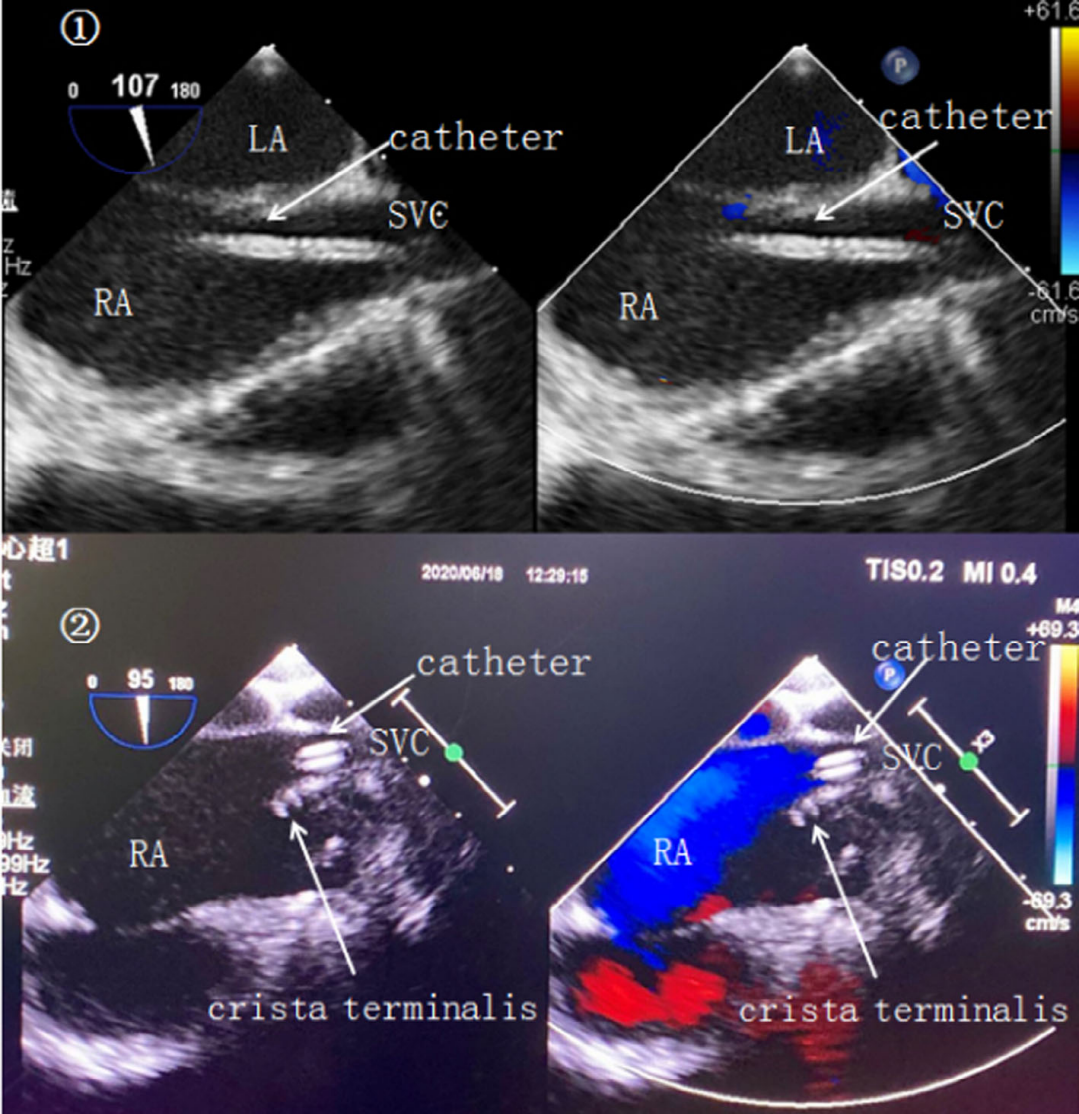
TEE images during catheter puncture and localization. (A) TEE image of the catheter entering the SVC inlet during the left subclavian vein puncture. (B) TEE image of the catheter tip finally positioned above the crista terminalis. RA, right atrium; SVC, superior vena cava; TEE, transesophageal echocardiography

A transesophageal ultrasound probe was inserted into the esophagus of the patient, and dynamic images of the heart and entrance of the RA were obtained through the posterior wall of the esophagus according to international guidelines.^14^ All venous access ports were Braun products (5‐F epoxy resin catheter; port weight, 3 g). Left SCV puncture was performed first using a sterile Seldinger technique, and the puncture point was located below the middle and outer 1/3 of the left clavicle. Then, the needle was advanced at a shallow angle (less than 30), and puncture was performed with negative pressure along the line connecting the coracoid process and the upper edge of the ipsilateral sternal bone of the clavicle toward the clavicle and first rib space.^6^ All ports and puncture supplies were rinsed in 10 U/ml heparin saline. After confirming the smooth return of blood, the guide wire was inserted into the left SCV. After confirming the presence of the guide wire in the RA under real‐time TEE visualization (Video 1), a 2‐mm incision was made at the puncture point, and the vascular expansion sheath was inserted. Then, a radiopaque silicone catheter was inserted into the vessel, and the guide wire was removed. TEE showed several parallel hyperechoic lines in the SVC or the RA (Figure 1) and was then used to locate the tip of the catheter at the SVC‐RA junction, defined as the superior edge of the crista terminalis (Video 2). The tip of the catheter is safe in the SVC within 1 cm of the SVC‐RA junction^12^ (Figure 1). The tip was also identified by the observation of many hyperechogenic microbubbles quickly flowing out of the distal catheter after a rapid flush of saline. If left SCV puncture or catheterization failed, right SCV puncture was performed. If right SCV puncture failed, the right or left internal jugular vein was selected for puncture and catheterization.^6^ The catheter length was recorded as the TEE‐derived length. Then, the extra catheter tubing was cut off, the injection seat was connected to the catheter, and the injection seat was placed in a subcutaneous pocket. Finally, the location of the catheter tip was verified by TEE. Intraoperative transesophageal examinations were performed by experienced cardiac anesthesiologists with expertise and qualifications in TEE. During the operation, TEE was used to guide the guide wire and to locate the tip of the catheter for all children (n = 36) involved in this study.

### 2.3 Catheter tip localization by chest X‐ray

Conventional chest X‐ray was used to locate the tip of the TIVAP catheter. All of the children (n = 36) who enrolled in this study underwent chest X‐ray to confirm the projection of the catheter tip. According to previous studies, when the X‐ray projection of the catheter tip is located between T5 and T7, the catheter tip is at the SVC‐RA junction.^15^

### 2.4 Catheter length calculated by height

Previous studies have shown that the length of a CVC can be calculated by the patient's height or external landmarks according to different puncture paths.^16^, ^17^, ^18^, ^19^ According to different puncture paths, formulas for calculating the depth of CVC catheterization using the patient's height have been put forward.^20^, ^21^ The formula for calculating the depth of CVC puncture through the left SCV puncture path is as follows: length of catheter (cm) = height of patient (cm)/10 + 2. The CVC length is the same as the TIVAP catheter length, as the tip of both catheters is placed at the SVC‐RA junction. Therefore, we used this formula to calculate the TIVAP catheter length, which is referred to as the height‐derived length.

### 2.5 Data collection and analysis

After surgery, the TIVAP could be used immediately. The surgical details and major complications during in‐hospital follow‐up after TIVAP surgery were collected through the hospital electronic medical records (EMR) system and the hospital information system (HIS). Close attention was paid to the occurrence of complications in patients after surgery, such as surgery‐related complications (e.g., hemothorax, pneumothorax), catheter twisting, blockage or leakage, and peri‐port skin infection, among others.

Statistical analyses were conducted using SPSS version 25.0. All data were tested using the Shapiro–Wilk test for normality and were verified for completeness. We used the Mann–Whitney U test to compare the height‐derived length and the TEE‐derived length. Summary descriptive statistics included the frequency (%), mean ± SD and median (IQR). p < .05 was considered statistically significant.

### 2.6 Sample size

The necessary sample size calculation was based on the paired rate comparison design. In our preliminary trial including 32 patients (unpublished data), the success rate of catheter tip localization was 100% which is P0 for TEE 81.25% which is P1 for chest X‐ray (T5‐T7). P2 is the difference between TEE and chest X‐ray success rates. P2 = P0‐P1 = 0.8125–1 = −0.1875. We applied PASS 11.0 to calculate that 29 patients would provide 90% power in detecting a difference (P2 = ‐0.1875) of −0.1875 using a two‐sided binomial test. The target significance level was 0.05. The actual significance level achieved by this test was 1.00. These results assume that the population proportion under the null hypothesis is 1.00. In consideration of the potential dropout rate (approximately 20%) and potential methodological problems that might be detected during the evaluation (i.e., change in puncture angle, or inadequate image quality on ultrasonography), we increased the sample size to 36.

## 3 RESULTS

Thirty‐six patients aged 5 months to 10 years old were studied. All of them underwent successful TIVAP placement through the left SCV. During the operation, no other positioning tools, such as bedside X‐ray, were used, and no complications, such as hemopneumothorax, occurred. Table 1 shows the basic characteristics and the catheter length measurements in the 36 patients.

**TABLE 1:** Basic characteristics in 36 patients

Characteristics	Results
Patients (n)	36
Males (%)	17 (47.22)
Age (months)	27 (12.25, 40)
Weight (kg)	12.25 (9, 14)
Follow‐up period (months)	6 (5.2, 6.8)
Disease	
Hematological malignancies (%)	20 (55.56)
Extracranial malignant solid tumor (%)	15 (41.67)
Nonmalignant tumor (%)	1 (2.78)
Complications	
Skin Infection around the port (n)	1

*Note:* Values are expressed as percentage, median (interquartile range).

**FIGURE 2:**
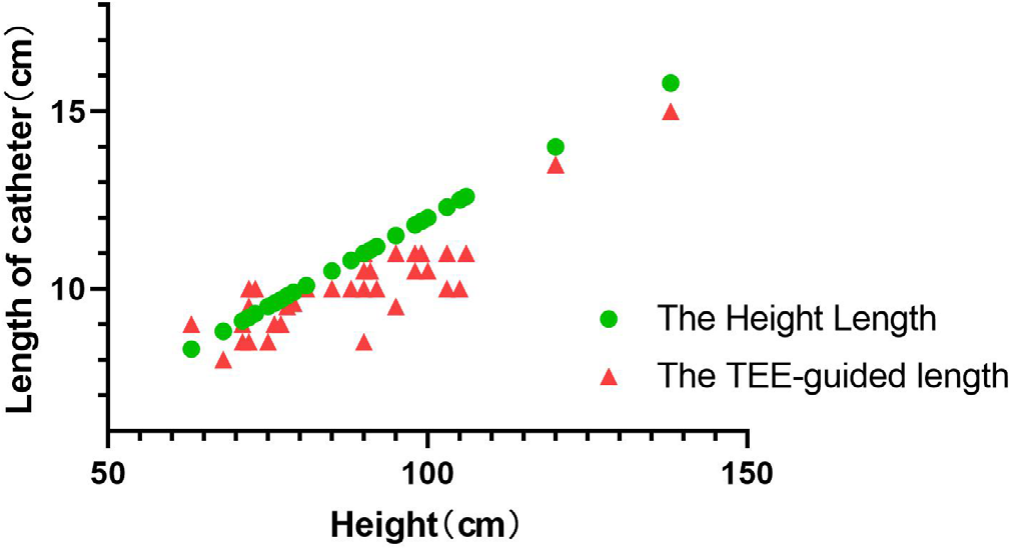
Distribution of catheter length (the height length and the TEE‐guided length) in scatter plot. TEE, transesophageal echocardiography

The success rate of TIVAP implantation was 100% in all enrolled patients, and the TIVAP could be used immediately. During the postoperative follow‐up period, one patient developed a skin infection around the port. No cases of pinch‐off syndrome, catheter dislocation, catheter twisting or catheter thrombosis were found in these patients. The tip of the catheter was successfully positioned at the SVC‐RA junction under TEE guidance in all 36 children, for a success rate of 100%. The height‐derived catheter length (11.0 [9.6, 11.8]) cm and the TEE‐derived catheter length (10.0 [9.3, 10.8]) cm were significantly different (p < .001) (Figure 2). The range of difference value of the height‐derived length and the TEE‐derived length is −0.8 to 2.5 cm(0.772 ± 0.8). The distribution of the tip position on chest X‐ray is shown in Table 2. Compared with TEE, chest X‐ray showed a coincidence rate of 80.56% in correctly detecting the TIVAP catheter tip locate. And seven children had a higher catheter tip located at T4, than the TEE location.

**TABLE 2:** Distribution of the tip position in the thoracic vertebral plane and the coincidence rate of the chest X‐rays and TEE in 36 patients

Thoracic	Patients n/%
T4	7/19.44%
T5	13/36.11%
T6	15/41.67%
T7	1/2.78%
Coincidence rate	29/80.56%

**FIGURE 3:**
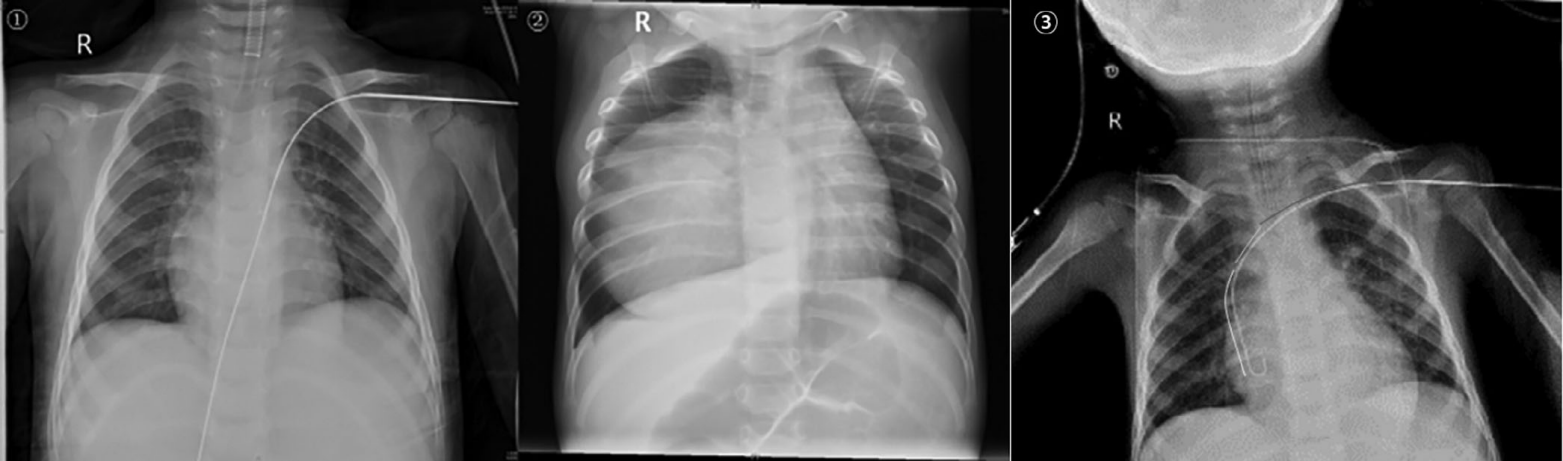
(A) Chest X‐ray of PLSVC child, the guide wire through the right atrium into the inferior vena cava, unable to locate the accurate place. (B) Chest X‐ray of the child with mediastinal tumors could not see the projection of the heart and could not accurately identify the position of the guide wire in the right atrium. (C) Pathway of guide wire in children with normal cardiac anatomy. The red line is the part of the guide wire in the left SCV section. The green line is the part of the guide wire in the SVC section. The yellow line is the part of the guide wire in the RA section. PLSVC, persistent left superior vena cava; RA, right atrium; SCV, subclavian vein; SVC, superior vena cava

We found that heart shadow could not be observed in some children with mediastinal tumors and the guide wire directly entered the inferior vena cava through the heart in children with persistent left superior vena cava (PLSVC) on chest X‐ray, which unable to locate the accurate routine of the guide wire (Figure 3). However, chest X‐ray could clearly show the guide wire passing through the left SCV, and then through the SVC, and finally entry into the RA in children with normal cardiac vascular anatomy (Figure 3).

## 4 DISCUSSION

TEE can provide clear images of SCV and RA through the posterior wall of the esophagus and can be used to measure distances accurate to the millimeter by Doppler imaging.^14^ We used TEE to locate the guide wire and catheter and finally verify the position of the catheter tip at the SVC‐RA junction. The success rate of TIVAP placement under TEE guidance in all patients was 100%, which is consistent with other studies.^12^ Generally, a TIVAP can be implanted and used for a long period of more than six months. Therefore, proper catheter placement is critical for the normal TIVAP use. During the postoperative follow‐up, no patients developed catheter blockage or drug leakage. One patient developed a skin infection around the port. This is probably related to the disinfection norms of TIVAP use postoperation and the reduced granulocyte levels in these children.

TEE guidance of TIVAP placement can facilitate the treatment of some special children. PLSVC is due to the failure of left SVC degeneration during the embryonic period.^22^ The left SVC flows through the dilated coronary sinus to the right atrium. Its incidence is 0.3–0.5% in the general population and 2–4% in congenital heart disease patients.^23^ The presence of anomalous vessels and abnormal intracardiac anatomy may lead to abnormal passage of the guide wire and it is difficult to judge the route of the guide wire by X‐ray^.^ ^24^ In some children with mediastinal tumor, the heart shadow cannot be observed on X‐ray. As a result, the location of the catheter tip can be difficult to determine directly in these special children through X‐ray. TEE can provide two‐dimensional images of the guide wire in the correct vessel and can be used to locate the catheter tip. We can use the TEE to obtain a plane at the junction of SVC and RA to confirm that the guide wire enters the RA in children with PLSVC. We can get images of their cardiac anatomy by TEE for children whose heart shadow cannot be seen through the chest X‐ray. Under the monitoring of TEE, the process that guide wire is from SVC to RA can be observed dynamically so that the catheter can be inserted smoothly. At the same time, we can also confirm the wire enters into the SVC correctly to avoid repuncture. However, X‐ray cannot achieve such visualization, let alone be used to guide and locate the catheter in these patients. In addition, radiation has potential adverse effects on children, especially children with cancer and hematological diseases. Children have a small body and thin tissue, and multiple exposures to radiation can lead to thyroid and breast cancer^.^ ^25^ As children and their families do not need to spend more time and energy for the collection of multiple X‐rays during operation and postoperative follow‐up, they are more satisfied with the use of intraoperative TEE.

The plane of the SVC and the entrance of the right atrium are readily observable by TEE with great accuracy. A number of studies have explored the relationship between height and the catheter length.^21^, ^26^ We found that the height‐derived catheter length and the TEE‐derived catheter length were significantly different (p < .001). The formula for calculating the length of a CVC and the catheter of TIVAP is relatively rough. Height is important when determining the length of the catheter of a CVC or a TIVAP, but the effect of physical characteristics (i.e., obesity) and the puncture point on the length of a SVC catheter can be biased. TEE guidance for positioning during surgery could save time and effort by avoiding the need to measure the height of the child before the operation.

In addition, in the conventional use of chest X‐ray to determine the position of the TIVAP catheter, multiple exposures are needed for confirmation. The coincidence rate of TEE and chest X‐ray regarding the catheter tip location was 80.56%. Seven children had a higher catheter tip located than the TEE location. However, if the catheter tip is located only by chest X‐ray, the catheter could end up being too long, the catheter will be placed in the heart, which may lead to thrombosis. At the same time, the use of chest X‐ray to locate the catheter tip is largely affected by the shooting angle, the anatomical location and breathing. A catheter in the SVC on the frontal projection may be outside the vascular space or may enter the azygous vein or the brachiocephalic vein in the posterior path.^26^ In fact, the catheter length is not very long in children, and chest X‐rays are focused on the range of T5–T7, which is too large of a range. Using standard body surface markers to estimate the correct catheter length for placement often results in ectopic catheterization.^10^, ^11^ This greatly increases the likelihood of intraoperative complications, such as pneumothorax, hemothorax, and even cardiac puncture.^2^, ^3^, ^4^, ^5^ We used TEE to guide the whole process and could accurately locate the SVC‐RA junction in every single patient.

In this study, no cases of pneumothorax or hemothorax occurred. After confirming that the guide wire was in the SVC or the RA by TEE, the vascular expansion sheath was inserted, and the catheter was placed. If the guide wire or the needle was inserted in the subclavian artery or the thoracic cavity accidentally, we could not identify the guide wire in the SVC or RA on TEE, and we immediately stopped misinsertion of the vascular expansion sheath to avoid fatal hemopneumothorax. We removed the supplies for the first time, and the surrounding tissue could close the puncture cavity because the needle and the guide wire were thin. Under the guidance of TEE, we can effectively avoid pneumothorax or hemothorax. The traditional puncture process determines the veins and arteries by the color of the blood. However, some children's venous blood is bright red; in some cases, higher pressures cannot exclude entrance of the guide wire into arteries or veins. The vascular expansion sheath was not inserted if the guide wire could not be identified in the SVC or RA in the two‐dimensional TEE image, which allowed the occurrence of hemopneumothorax to be effectively avoided.

However, we acknowledge the limitations of TEE in TIVAP catheter guidance and localization. In some practice patterns, surface guided subclavian ultrasound may be performed adjunctively or instead of the other methods. We have no data comparing this specific method because its use was difficult in children. Considering the occlusion of the clavicle in children, it was difficult to puncture the SCV under surface ultrasound guided. However, our blinded puncture experience is comparable to others way very low, comparable complications rates. This blinded approach remains a widely used alternative in Europe, America, Japan, Singapore, and parts of China.^27^, ^28^, ^29^, ^30^, ^31^ Furthermore, we had no data comparing TEE with TTE, because we found that using trans‐thoracic echocardiography to find the position of the crista terminalis, it was easy to be affected by the angle of ultrasonic probe, lung and liver, resulting in errors. In addition, the operating scope of trans‐thoracic echocardiography is covered by aseptic towels and may even cross the aseptic area of the operation, and this can be limited by surgical sterile procedures. Furthermore, TEE equipment is expensive and may not be available in many primary hospitals. The scope of visualization provided by the TEE probe is limited, and the direction of the guide wire cannot be observed. In left SCV puncture, the guide wire may go to the right side or toward the cranial side of the blood vessel. An experienced surgeon can try to avoid such problems. If the position of the guide wire cannot be verified during the operation or the TEE probe does not show the guide wire in the SVC or RA, bedside X‐ray examination can be applied to confirm the direction of the guide wire.

## 5 CONCLUSION

Our findings suggest that the use of TEE in TIVAP implantation could ensure accurate placement of the catheter in the SVC or RA and correct localization of the catheter tip. The TEE positioning results are more accurate than height calculation formula and chest X‐ray. The technique we use can avoid intraoperative complications such as hemopneumothorax and arterial injury. TEE provides a new and accurate method for TIVAP placement and positioning in children.

## CONFLICT OF INTEREST

Author Shujun Yang declares that she has no conflict of interest. Author Xiangru Kong declares that he has no conflict of interest. Author Lifei Liu declares that he has no conflict of interest. Author Ying Xu declares that she has no conflict of interest. Author Jun Zhang declares that he has no conflict of interest.

## PATIENT CONSENT STATEMENT

Prior written informed consent was provided by the child or the child's guardians. The child or child's guardian signed the informed consent and participated in this study.

## Supporting information


**Appendix S1** Supporting information.

**Video 1** Guide wire in right atrium: The puncture guide wire shows hyperechoic development in real‐time TEE images, as shown in the video. We can make sure that the guide wire enters the right atrium, not other blood vessels or tissues. After that, we insert the vascular expansion sheath and catheter safely to prevent from damaging blood vessels and causing fatal hemopneumothorax.

**Video 2** Location of catheter tip: We confirm that after the guide wire enters the right atrium, the vascular expansion sheath and catheter are inserted. At TEE real‐time two‐dimensional images, the catheter shows a special “double‐track” sign, as shown in the video. In order to place the tip of the catheter within what we think is safe; we place the tip within 1 cm of the SVC‐RA junction, defined as the superior edge of the crista terminalis.

## Data Availability

The data used to support the findings of this study are available from the corresponding author upon request.
