# Supplementary material for: Application of transesophageal echocardiography for localization in totally implantable venous access port implantation through subclavian approach in children
Source: Clin Cardiol. 2020 Nov 25;44(1):129–35. doi: 10.1002/clc.23518 (PMC7803355; doi:10.1002/clc.23518)
Supplement: Supplementary file 1 — Appendix S1 Supporting information. [file CLC-44-129-s001.zip › CLC_23518_Informed Consent for Clinical Medical Research (Children's Edition).docx]

**Informed Consent for Clinical Medical Research (Children's Edition)**

**Application of** **transesophageal echocardiography for localization in** **totally implantable venous access port implantation through subclavian approach in children**

Dear children:

We are considering inviting you to participate in a study on the application of transesophageal echocardiography in catheter tip localization in pediatric totally implantable venous access port implantation. Before agreeing to participate in the study, it is important that you understand the details of the study. Please read this document carefully and ask questions. This study has been approved by the Ethics Committee of Medical institutions. Whether to participate in this study depends entirely on your personal wishes.

**1）The purpose of the study**

The main purpose of this study was to explore the localization effect of transesophageal echocardiography in children's totally implantable venous access port implantation. By comparing the success rate of transesophageal echocardiography and traditional chest X-ray in locating the catheter tip of totally implantable venous access port, the success rate of infusion port implantation in children can be improved, and the related complications can be reduced as far as possible.

**2）An explanation of the research**

Totally implantable venous access port is a vascular channel device for repeated puncture, which can be used for a long time or intermittently. Totally implantable venous access ports are widely used in patients who need long-term intravenous administration, especially cancer patients who need long-term chemotherapy. Conventional, people usually used chest X-ray to locate the tip of the catheter. With the progress of medicine and the deepening of understanding, transesophageal echocardiography has been gradually used to guide totally implantable venous access port implantation in recent years, without radiation damage, and can accurately locate the tip position of catheter and improve the accuracy of positioning. American Andropoulos DB et al. published a controlled study on transesophageal echocardiography guiding central venous catheterization in patients with congenital heart surgery, indicating that the success rate of transesophageal echocardiography guided deep venous catheterization was 100%.

Therefore, we intend to compare the localization effect of transesophageal echocardiography and chest X-ray on the tip of catheter in totally implantable venous access port implantation with reference to the research plan of Andropoulos DB et al., and to explore the localization method suitable for China's national conditions. Through this study, we hope to find out the possibility of improving the accuracy of catheter tip positioning in Chinese children in infusion port implantation, and the occurrence of related complications. In order to improve the success rate of totally implantable venous access port implantation in Chinese children, reduce the related complications as far as possible, and provide a theoretical basis for the location of catheter tip in the future transesophageal echocardiography in totally implantable venous access port implantation.

**3）The number of subjects and duration of patient participation**

About 50 pediatric patients requiring totally implantable venous access port implantation will participate in the study. The total time of this study is estimated to be 24 months, and your participation time is estimated to be 6 months.

**4）The procedures to be followed by patients**

This study will collect the medical information data generated by you in the routine clinical diagnosis and treatment, and the imaging data of the operation process during the operation. We will carry out experimental research and analysis on these data to explore the localization of transesophageal echocardiography in children's totally implantable venous access port implantation. If the operation is successful, and you need to come to the oncology clinic for review ,on 14 days ,28 days ,42 days ,56 days after surgery. According to the routine requirements of totally implantable venous access port implantation patients after long-term follow-up.

After you understand the content of the whole study and your questions have been answered satisfactorily, if you are willing to participate in this study, you need to sign this informed consent.

The researchers began to arrange for related examinations and research operations. These inspection and research operations will help determine whether you are suitable to participate in this study. This stage is "pre-study stage ". If the researchers determine that you do not meet the requirements for intraoperative catheter tip positioning using transesophageal echocardiography, you will not be allowed to participate in this study. The researchers will recommend that you follow the conventional totally implantable venous access port catheter tip positioning program.

**5）The potential discomforts and risks**

Possible risks and discomfort in this study include:

Transesophageal echocardiography related risk: esophageal injury, very unlikely, the vast majority of stop operation can stop bleeding. The risk of totally implantable venous access port implantation belongs to the risk of surgical treatment and has been specified in the informed notification of treatment.

There are no other risks in collecting information data and scrapping the organization.

**6）The expected benefits**

Your participation in this study may improve the positioning accuracy of the tip of your totally implantable venous access port catheter and postoperative comfort, and may provide a theoretical basis for helping other patients in the future.

**7）The alternative treatments and procedures that might also be beneficial**

You do not have to participate in this study to receive totally implantable venous access port implantation. Besides participating in this study, you can also choose traditional chest X-ray positioning totally implantable venous access port implantation.

**8）The compensation or medical treatments available if injury occurs**

If you choose chest X-ray or transesophageal echocardiography in operation , it may take longer operation time, the cost of treatment may increase, we will not give you any subsidy. At the same time, you will not be paid for this study.

If you have suffered research-related damage during your participation in the study, we will provide you with emergency treatment free of charge. Damage compensation during the study will be carried out in accordance with our GCP and other relevant laws.

**9）** **The protection of confidentiality and privacy**

The researcher is responsible for following applicable data protection regulations to process your research data. However, the information can be accessed by the Ethics Committee and the superior administration. The results may be published in medical journals / conferences, but your personal information will not be made public.

Your health information is protected by relevant Chinese laws. After signing this informed consent, it indicates that you agree to collect, use and share your health information data with research doctors and research center personnel. Your initials and a code assigned to the research data are now available to the researcher. Your authorization to allow us to use your health information remains valid until the end of the study and until the results are available. After the study is finished and the results are obtained, we will delete your personal information from the research record.

**10）The participation is voluntary**

Participation in this study is entirely out of your personal will. You can choose not to participate in this study, you can also withdraw freely at any time. Any medical treatment and rights will not be affected or discriminated against by medical personnel.

**11）Who to contact with questions about the research**

All members of the study group will answer all your questions before you sign this consent. If you still have questions, suggestions or opinions after signing the consent, you can also communicate with the researchers. You can keep abreast of the information and progress of this study.

Researcher and contact number: Jun Zhang, M.D. 13983460980

12）**The information of sponsor**

The study will be conducted in Department of Surgical Oncology, Children's Hospital of Chongqing Medical University. The lead researcher is Jun Zhang M.D., and other research members.

**A statement that participation is voluntary**

The research doctor or related researchers have verbally informed me of the information in this study, and I have read the above written information.

I have been given ample opportunity to discuss and ask questions about the above research.

I agree to participate in this study and understand that my participation in the study is entirely voluntary.

I understand that I can withdraw from the study at any time, and my withdrawal will not affect my future medical treatment.

To sign this informed consent, I agree that my personal information data, including my medical information data, will be used in the above manner.

I know i will get a copy of this informed consent.

Signature of legal guardian Date _____________________

Relationship with patients_________________ Telephone numbers ___________________

Statements of researcher :

I will strictly abide by the obligations stipulated in Article 22 of the Law of the People's Republic of China on Professional Physicians; abide by laws, regulations and technical operating norms; establish professionalism, abide by professional ethics and fulfill my duties in serving patients; care for, love and respect patients and protect their privacy; work hard to study business, update knowledge and improve professional and technical level; publicize health care knowledge and carry out health education for patients.

Signature of researchers Date

Telephone numbers _______________________________
