# Supplementary material for: Application of transesophageal echocardiography for localization in totally implantable venous access port implantation through subclavian approach in children
Source: Clin Cardiol. 2020 Nov 25;44(1):129–35. doi: 10.1002/clc.23518 (PMC7803355; doi:10.1002/clc.23518)
Supplement: Supplementary file 1 — Appendix S1 Supporting information. [file CLC-44-129-s001.zip › CLC_23518_IRB.pdf]

重庆医科大学附属儿童医院伦理委员会审查批件

Approval Letter of the Institutional Review Board, Children's Hospital of Chongqing Medical University

批件号（File No.）：（2019）年伦审（研）第（230）号

项目名称 Protocol name	经食道超声心动图在儿童输液港植入术中定位的应用		
申办单位 sponsor	重庆医科大学附属儿童医院		
承担单位 Institute	重庆医科大学附属儿童医院		
项目类别 Protocol Type	1. 药物：☐Ⅰ期 ☐Ⅱ期 ☐Ⅲ期 ☐Ⅳ期<br>2. 器械：☐Ⅰ类 ☐Ⅱ类 ☐Ⅲ类 3. ☐试剂 4. ☑其它		
申请科室 Application Department	肿瘤外科	项目负责人 Principal Investigators	章均
审查日期 Date Reviewed	2019.11.28	批准日期 Date Approved	2019.11.29
审查方式 Means of Reviewing	☐会议审查（☐初审 ☐复审）<br>☑快速审查（☐初审 ☑复审）		

审查意见 Decision：

医学研究伦理委员会 2019 年 11 月 20 日对该项目进行了会议审查，结论为“作必要修正后同意”，项目修正后提交复审申请，快速审查结论为“同意”。请严格按照方案开展临床研究项目。

跟踪审查频率 Frequency of Tracking Review：

☐否 ☑是：☐3 个月 ☐ 6 个月 ☐9 个月 ☑12 个月

审查结论 Conclusions： 同意

有 效 期 Valid Period： 2019-11-29 至 2021-11-1

注意事项（请仔细阅读）：

1. 已批准项目应遵循本伦理委员会批准的方案执行，需符合《涉及人的生物医学研究伦理审查办法》（中华人民共和国国家卫生和计划生育委员会令第 11 号）、SFDA《药物临床试验质量管理规范（2003）》、《医疗器械临床试验质量管理规范》（国家食品药品监督管理总局、中华人民共和国国家卫生和计划生育委员会令第 25 号）、WMA《赫尔辛基宣言》和 CIOMS《人体生物医学研究国际道德指南》的伦理原则。
2. 研究过程中若变更主要研究者，对临床研究方案、知情同意书、招募材料等的任何修改，请申请人提交修正案审查申请。
3. 发生严重不良事件，请申请人及时提交严重不良事件报告。
4. 请按照伦理委员会规定的跟踪审查频率，在跟踪审查日到期前 1 个月提交研究进展报告。
5. 方案违背/偏离，暂停/提前终止临床研究，需及时通知本伦理委员会。
6. 研究结束时，须向本伦理委员会提交结题报告。

重庆医科大学附属儿童医院医学研究伦理委员会（盖章）

主任委员：[illegible]

2019 年 11 月 29 日

申明：本伦理委员会的组成及工作程序符合中国食品药品监督管理局颁布的“药物临床试验质量管理规范”（2003 年）中的相关要求及其所遵循的 ICH GCP 指导原则。

附：
